# RhoA/Rho-Kinase and Nitric Oxide in Vascular Reactivity in Rats with Endotoxaemia

**DOI:** 10.1371/journal.pone.0056331

**Published:** 2013-02-15

**Authors:** Mei-Hui Liao, Chih-Chin Shih, Cheng-Ming Tsao, Shiu-Jen Chen, Chin-Chen Wu

**Affiliations:** 1 Graduate Institute of Medical Sciences, National Defence Medical Centre, Taipei, R.O.C., Taiwan; 2 Department of Pharmacology, National Defence Medical Centre, Taipei, R.O.C., Taiwan; 3 Department of Anesthesiology, Taipei Veterans General Hospital, National Yang-Ming University, and National Defence Medical Centre, Taipei, R.O.C., Taiwan; 4 Department of Nursing, Kang-Ning Junior College of Medical Care and Management, Taipei, R.O.C., Taiwan; 5 Department of Physiology, National Defence Medical Centre, Taipei, R.O.C., Taiwan; University of Padova, Italy

## Abstract

RhoA/Rho-kinase (RhoA/ROK) pathway promotes vasoconstriction by calcium sensitivity mechanism. LPS causes nitric oxide (NO) overproduction to induce vascular hyporeactivity. Thus, we tried to examine the role of RhoA/ROK and NO in the regulation of vascular reactivity in different time-point of endotoxaemia. Male Wistar rats were intravenously infused for 10 min with saline or *E. coli* endotoxin (lipopolysaccharide, LPS, 10 mg/kg) and divided to five groups (*n* = 8 in each group): (i) Control, sacrificed at 6 h after saline infusion; (ii) LPS1h, sacrificed at 1 h after LPS infusion; (iii) LPS2h, sacrificed at 2 h after LPS infusion; (iv) LPS4h, sacrificed at 4 h after LPS infusion; and (v) LPS6h, sacrificed at 6 h after LPS infusion. LPS1h and LPS2h were regarded as early endotoxaemia, whereas LPS4h and LPS6h were regarded as late endotoxaemia. Indeed, our results showed that LPS reproduced a biphasic hypotension and sustained vascular hyporeactivity to noradrenaline (NA) *in vivo.* Interestingly, this hyporeactivity did not occur in *ex vivo* during early endotoxaemia. This could be due to increases of aortic RhoA activity (*n* = 5, *P*<0.05) and myosin phosphatase targeting subunit 1 phosphorylation (*n* = 3, *P*<0.05). In addition, pressor response to NA and vascular reactivity in early endotoxaemia were inhibited by ROK inhibitor, Y27632. Furthermore, plasma bradykinin was increased at 10 min (24.6±13.7 ng/mL, *n* = 5, *P*<0.05) and aortic endothelial NO synthase expression was increased at 1 h (+200%. *n* = 3, *P*<0.05) after LPS. In late endotoxaemia, the vascular hyporeactivity was associated with aortic inducible NO synthase expression (*n* = 3, *P*<0.05) and an increased serum NO level (*n* = 8, *P*<0.05). Thus, an increased RhoA activity could compensate vascular hyporeactivity in early endotoxaemia, and the large NO production inhibiting RhoA activity would lead to vascular hyporeactivity eventually.

## Introduction

Vascular reactivity is mainly associated with smooth muscle contractility which is dually regulated by cytoplasmic Ca^2+^ concentration and Ca^2+^ sensitivity. The pathway of RhoA/Rho-kinase (RhoA/ROK) is the major cellular target for regulating Ca^2+^ sensitivity of agonist-induced contraction (including α_1_-adrenergic agonist) [1], [2]. The activation of RhoA leads to stimulation of ROK that can phosphorylate and subsequently inactivate myosin light chain (MLC) phosphatase (MLCP), favoring MLC phosphorylation, actin-myosin interaction and cell contraction [3]–[5]. MLCP consists of a catalytic 38-kDa PP1c, an associated 110- to 130-kDa myosin phosphatase targeting subunit 1 (MYPT1) and a tightly bound 20-kDa subunit of unknown function [6]. MYPT1 is responsible for binding to PP1c and targeting myosin. Two phosphorylated sites of MYPT1 (at Thr696 and Thr850) have been proposed for the in situ inhibition of MLCP via G_α12/13_/RhoA/ROK pathway [7]–[11].

Previous studies have shown that protein kinase G (PKG) inhibits RhoA-induced Ca^2+^ sensitization by phosphorylating Ser188 of RhoA [12], [13]. In addition, the RhoA/ROK pathway is inhibited by cyclic guanosine monophosphate (cGMP) dependent mechanism after lipopolysaccharide (LPS) in small mesenteric arteries [14]. These indicate that cGMP/PKG pathway regulates the RhoA/ROK activity leading to vasodilation. Endotoxin (LPS) is a major component of the outer membrane of Gram-negative bacteria and one of the most potent microbial initiators of inflammation [15], [16]. Administration of rats with LPS causes nitric oxide (NO) overproduction that induces a biphasic hypotension (an early hypotension from 30 min to 2 h and a delayed hypotension from 4 h to 6 h after LPS in our rat model) [17], and causes a sustained attenuation to vasoconstrictor agents [18]–[20]. The early hypotension induced by LPS is proposed to be mainly associated with the release of endogenous bradykinin (BK), which activates endothelial NO synthase (eNOS), resulting in vasodilation. This is based on the fact that kininogen-deficient rats or kinin receptor-deficient mice reverse early hypotension occurred in endotoxaemic animals [21]–[23]. In addition, excessive NO production by inducible NO synthase (iNOS) participates in LPS-induced delayed hypotension and aortic hyporesponsiveness to noradrenaline (NA) [24], [25]. Thus, inhibition of NO synthesis could attenuate hypotension and vascular hyporeactivity to NA [26]. However, there is no *in vivo* evidence to explore the effect of RhoA/ROK in animals with endotoxaemia and the interaction of RhoA/ROK and NO in the regulation of vascular reactivity at different time-point in endotoxaemia *in vivo* and *ex vivo* experiments.

In this study, we administrated rats with LPS to induce endotoxaemia, and recorded the changes of haemodynamics, biochemical variables, pressor response to NA, RhoA activity, NO levels as well as BK levels at baseline, 1 h, 2 h, 4 h and 6 h after saline or LPS infusion, In addition, we evaluated vascular reactivity *ex vivo*. We tried to examine (i) the role of RhoA/ROK in the regulation of vascular reactivity and (ii) the relationship between RhoA/ROK and NO at different time-point of endotoxaemia.

## Materials and Methods

### Materials

LPS (*E. coli* serotype 0127:B8), NA, acetylcholine (ACh) and Y27632 were purchased from Sigma Chemical Co. (St Louis, MO, USA). Anti-iNOS, anti-eNOS, anti-RhoA, anti-total-MYPT1 and anti-β actin antibodies were purchased from BD Transduction Laboratories (Lexington, KY, USA). Anti- phospho-MYPT1-Thr^696^ antibody was purchased from Millipore (Billerica, MA, USA). Anti-phospho-MYPT1-Thr^850^ antibody was purchased from Upstate (Lake Placid, NY, USA). GTP-Linked Immunosorbant Assay (G-LISA, RhoA Activation Assays Biochem Kit) was purchased from Cytoskeleton (Denver, CO, USA). Bradykinin (BK) EIA Kit was purchased from Phoenix Pharmaceuticals Inc. (Burlingame, CA, USA).

### Animals and Experimental Procedures

**Figure 1:**
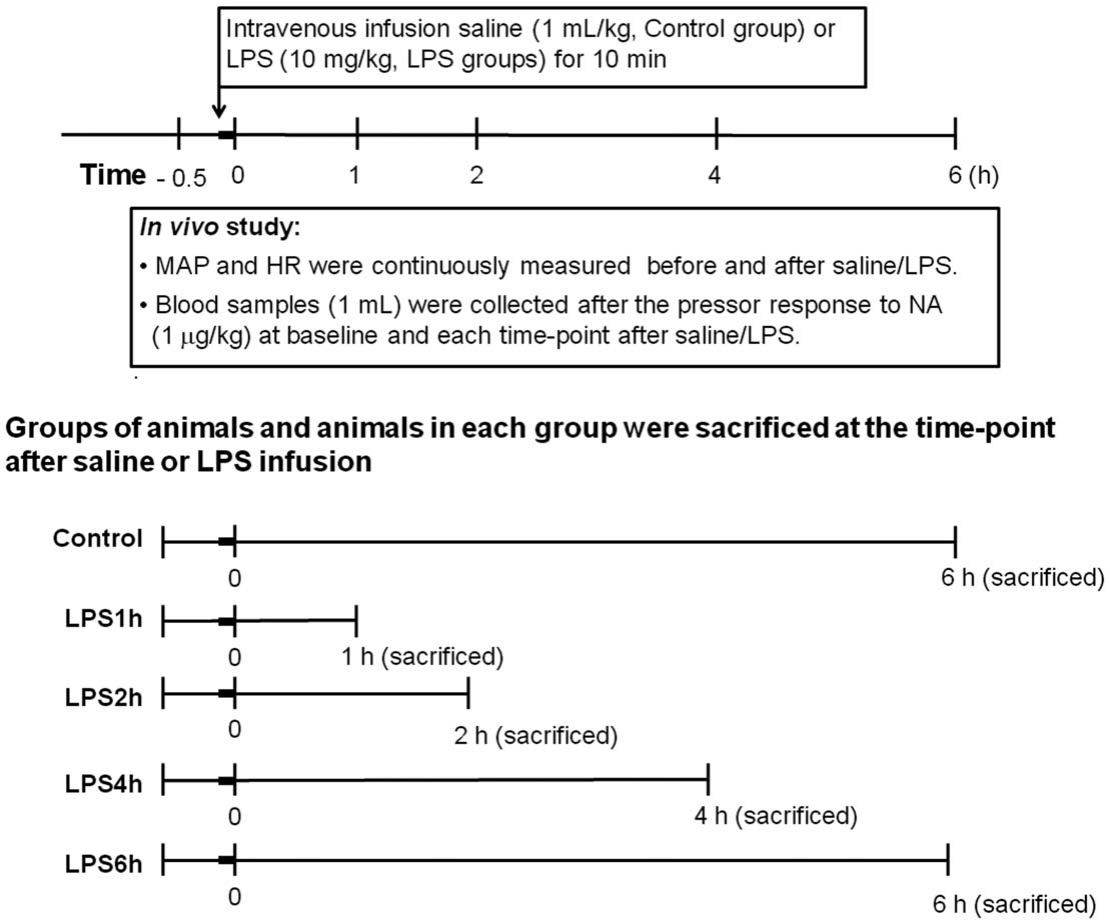
Experimental protocols are designed in this study. Wistar rats were divided to five groups: (i) Control, rats were sacrificed at 6 h after saline (0.9% NaCl infusion 10 min); (ii) LPS1h, rats were sacrificed at 1 h after LPS (lipopolysaccharide, LPS 10 mg/kg; infusion 10 min); (iii) LPS2h, rats were sacrificed at 2 h after LPS (10 mg/kg, infusion 10 min); (iv) LPS4h, rats were sacrificed at 4 h after LPS (10 mg/kg, infusion 10 min); (v) LPS6h, rats were sacrificed at 6 h after LPS (10 mg/kg, infusion 10 min).

All experimental procedures were approved by the institutional and local Committee on the Care and Use of Animals (National Defence Medical Centre, Taipei, ROC, Taiwan) (Permit Number: IACUC-10-199) and provided assurance that all animals received humane care according to the criteria outlined in the Guide for the Care and Use of Laboratory Animals prepared by the National Academy of Sciences. Male Wistar rats were purchased from BioLASCO Taiwan Co. (Taipei, ROC, Taiwan) and were guaranteed free of particular pathogens. All rats were bred and maintained under a 12 h light-dark cycle at a controlled temperature (21°C±2°C) with free access to standard rat chow and tap water. Male Wistar rats weighing 250–300 g were intraperitoneally anaesthetized with sodium pentobarbital (50 mg/kg). Polyethylene catheters were placed in the right jugular vein and left carotid artery and the distal end of the catheter was externalized through an incision in the back of the neck for measurement of haemodynamic and blood withdrawal. The cannulated animals were allowed to recover to normal condition overnight. Then, animals were intravenously infused saline (1 mL/kg) or LPS (10 mg/kg) for 10 min and randomly allocated into five groups (*n* = 8 in each group) as followed: (i) Control, sacrificed at 6 h after saline; (ii) LPS1h, sacrificed at 1 h after LPS; (iii) LPS2h, sacrificed at 2 h after LPS; (iv) LPS4h, sacrificed at 4 h after LPS; (v) LPS6h, sacrificed at 6 h after LPS (Fig. 1). Animals treated with LPS for 1 h or 2 h were regarded as having early endotoxaemia, and those treated for 4 h and 6 h were regarded as having late endotoxaemia. All haemodynamic and biochemical parameters were monitored during the experimental period in each group. Arterial blood samples (1 mL) were drawn prior to (i.e. at time 0) and after saline or LPS. The blood samples were replaced immediately by the injection of an equal volume of saline to maintain blood volume. At the end of each experiment, rats were sacrificed by intravenous administration of overdosed pentobarbital and thoracic aortas were obtained and cleared of adhering periadventitial fat for isometric tension experiment, Western blotting and RhoA activity assay. In a separate experiment, animals were randomly allocated into two groups (*n* = 5 in each group): (i) LPS group (LPS, 10 mg/kg, infusion 10 min); (ii) Y27632+LPS group (Y27632, 5 mg/kg, infusion 30 min; LPS, 10 mg/kg, infusion 10 min). In this study, we examined the effect of Y27632 on the changes of haemodynamics in rats with endotoxaemia.

In addition, this study required a large amount of aortas to perform the *ex vivo* experiments. Due to the regulation of 3R (replacement, reduction, and refinement) in using animals for study, we only used 3–5 rats in each group to achieve RhoA, total MYPT1, phosphorylated-MYPT1, eNOS, and iNOS protein expression, as well as RhoA activity in aorta *ex vivo*.

### Measurement of Mean Arterial Pressure (MAP) and Heart Rate (HR)

Measurement of MAP and HR were performed on pairs of conscious rats. The arterial catheter was connected to a pressure transducer (P23ID, Statham, Oxnard, CA, USA) for continuous measurement of MAP and HR before infusion of saline or LPS and until the animals were sacrificed (Fig. 1) on a multichannel recorder (MacLab/4e, AD Instruments Pty Ltd., Castle Hill, Australia).

### Determination of Vascular Reactivity *in vivo* and *ex vivo*

Baseline refers to a measurement made at a stage of control or endotoxaemia prior to NA administration. In the *in vivo* study, after recording of baseline haemodynamic parameters, a single bolus injection of NA (1 µg/kg, i.v.) was used to evaluate vascular responsiveness at that stage of sepsis (Fig. 1). At the end of the *in vivo* experiment, thoracic aortas were obtained from each group as described above. The vessels were cleared of adhering periadventitial fat and were cut into segments 2.5 mm in length. The rings were mounted in 20-mL organ baths filled with warmed (37°C), oxygenated (95% O_2_/5% CO_2_) Krebs’ solution (pH 7.4) [27]. Isometric force was measured with Grass FT03 type transducers (Grass Instruments, Quincy, MA, USA) and recorded on a MacLab Recording and Analysis System (AD Instruments Pty Ltd., Castle Hill, Australia). The rings were allowed to equilibrate for 60 min under an optimal resting tension of 2 g and the experimental protocols begun once the aortas had reached a steady basal resting tension. Briefly, NA (1 µmol/L) and ACh (1 µmol/L) were applied to establish control responsiveness. Then, concentration-response curves to NA (1 nmol/L –30 µmol/L) were obtained to evaluate the vascular reactivity in each group. In a separate experiments, aortic rings were treated with Y27632 (a ROK inhibitor) for 15 min before NA was added. The concentration-response curve of control (*n* = 7 in each concentration), LPS1h and LPS2h (*n* = 5 in each group) groups were performed in the presence of Y27632 (10 nmol/L –0.3 µmol/L in Control group, 0.1 µmol/L in LPS1h and LPS2h groups).

### Quantification of Organ Function

Whole blood was immediately centrifuged for 2 min at 7,000 *g* and 40 µL of the serum was taken to measure: (i) lactate dehydrogenase (LDH); (ii) alanine aminotransferase (ALT); (iii) blood urine nitrogen (BUN); and (iv) creatinine (CRE) (Fuji DRI-CHEM 3030, Fuji Photo Film Co., Tokyo, Japan). The serum was immediately stored at −20°C for subsequent measurement of nitrite/nitrate.

### Measurement of Serum Nitrite/Nitrate Level

Nitrite and nitrate are the primary oxidation products of NO and therefore the nitrite/nitrate level in serum can be regarded as an indicator of NO formation. Thirty microlitre serum stored at −20°C were thawed and de-proteinized by incubating them with 95% ethanol (4°C) for 30 min. The samples were subsequently centrifuged for an additional 5 min at 14,000 *g*. The NO concentration in all samples was measured by using chemiluminescence. In this method, nitrate is reduced to NO via nitrite. The amount of nitrate in serum (6 µL) was measured by adding a reducing agent (0.8% VCl_3_ in 1N HCl) to the purge vessel to convert nitrate to NO, which was stripped from the serum by using a helium purge gas. The NO was then drawn into the Sievers Nitric Oxide Analyzer (Sievers 280 NOA, Sievers Inc., Boulder, CO, USA) [28].

### Western Blot Analysis

At the end of *in vivo* experiment, thoracic aortas were obtained from each group as described above. Protein concentration was determined by BCA Protein Assay Kit (Thermo scientific, Rockford, IL, USA). Samples containing 90–150 µg of protein were processed for analysis. Protein was subjected to 10 or 12% sodium dodecyl sulfate-polyacrylamide gel electrophoresis under reducing condition. The protein was transferred onto nitrocellulose membranes (Mini Trans-Blot Cell, Bio-Rad Laboratories, Hercules, CA, USA). The membranes were blocked with 5% non-fat milk in Tris buffer solution containing 0.1% Tween-20 (TBST) for 1.5 h at room temperature. The membranes were then incubated overnight at 4°C, with gentle shaking with primary antibody (iNOS, 1∶1000 dilution; eNOS, 1∶500 dilution; RhoA, 1∶1000 dilution; total-MYPT1, 1∶1000 dilution; β-actin, 1∶10000 dilution; phospho-MYPT1 at Thr696, 1.5 µg/mL; and phospho-MYPT1 at Thr850, 1.5 µg/mL) in TBST buffer with 3% milk. The membranes were washed and then incubated at room temperature with horseradish peroxidase-conjugated goat anti-mouse IgG (1∶3000 dilution, BD Transduction Laboratories, Lexington, KY, USA) or horseradish peroxidase-conjugated goat anti-rabbit IgG (1∶3000 dilution, Cell signaling Technology Inc, Danvers, MA, USA) in TBST buffer. The membranes were washed and proteins were visualized by using an enhanced peroxidase/luminal chemiluminescence reaction (ECL Western blotting detection reagent) (Thermo scientific, Rockford, IL, USA). Bands were quantified by densitometry using UVP image software (UVP, Upland, CA, USA).

### Measurement of RhoA Activity

RhoA activity was analyzed by using the GTP-Linked Immunosorbant Assay (G-LISA, RhoA Activation Assays Bicochem Kit) according to the manufacturer’s instructions. Briefly, previously stored samples were homogenized in lysis buffer and then centrifuged at 18,000 *g* for 2 min at 4°C. The supernatant, containing 30 µg protein, and an equal volume (60 µL) of ice-cold binding buffer were added into each well of the 96-well plate containing protein samples. After plates were shaken on a cold orbital microplate shaker for 30 min at 4°C, the solution was removed from the wells and the plates were incubated with diluted anti-RhoA primary antibody (1∶250), followed by secondary antibody (1∶62.5), on a microplate shaker at room temperature for 45 min. The solution was removed from the wells and the plates were incubated with horseradish peroxidase detection reagent for 15 min at 37°C. After the addition of horseradish peroxidase stop buffer, the absorbance was read immediately at 490 nm.

### Measurement of BK Level in Plasma

Plasma BK level was analyzed in triplicate by using the competitive enzyme immunoassay system (Bradykinin EIA Kit, Burlingame, CA, USA) according to the manufacturer’s instructions. Briefly, blood samples were collected in tubes containing EDTA (1 mM) and aprotinin (0.6 TIU/ml) and were gently rocked to inhibit the activity of proteinases, and then centrifuged at 1600 *g* for 15 min at 4°C and 1 mL plasma was collected. The 1 mL plasma added an equal amount of buffer A to acidify the plasma, and then centrifuged at 12000 *g* for 20 min at 4°C. The acidified plasma was loaded onto the pre-equilibrated SEP-Column containing 200 mg C18. Slowly washed the column with buffer A and discarded the wash. Eluted the peptide with buffer B and collected the eluent into a polystyrene tube and then to dryness in a centrifugal concentrator. Pellet was stored at −20°C until further use. Rehydrated samples with 50 µL of assay buffer, and then added samples, primary antibody and biotinylated peptide of 50 µL in turn into each well except for blank. After immunoplate were shaken for 2 h at room temperature, the solution was removed from the well and washed 4 times. After the immunoplate were shaken for 1 h at room temperature, added 100 µL of streptavidin-horseradish peroxidase that catalyzed the substrate solution into each well, and then repeated above wash step. Added 100 µL of TMB substrate solution incubated 1 h at room temperature. After adding 2 N HCl into each well to stop the reaction, absorbance was read immediately at 450 nm. BK concentration was assessed by extrapolation of the standard curve made from dilutions of known BK concentration.

### Statistical Analysis

Results are expressed as mean ± standard error of mean of *n* determinations, where *n* represents the number of animals studied. Statistical significance was determined through one-way analysis of variance followed by Student-Newman-Keuls test. A *p* value <0.05 was considered statistically significant.

## Results

### Time-course Changes of Haemodynamic Parameters and Organ Functions

The basal levels of haemodynamic and biochemical variables as well as serum NO were not significantly different among experimental groups studied (Table 1). The animals in LPS groups showed significantly progressive increases in HR, ALT, CRE, BUN, LDH and NO during the experimental period (*n* = 8 in each groups, *P*<0.05), whereas LPS caused a biphasic change on blood pressure, including an immediate and transient fall in mean arterial pressure (MAP) (∼60 mmHg at 30 min) and a sustained decline from 2 h (101±3 mmHg) to 6 h (68±4 mmHg), which was accompanied by a significant increase in HR during the experimental period (from 339±18 to 561±16 beats per minute, *P*<0.05). However, in the control group, there was no significant change in haemodynamic, biochemical parameters and serum NO levels during the experimental period (Table 1).

**Table 1:** Changes of haemodynamic and biochemical parameters in control and lipopolysaccharide (LPS) groups.

	0 h	1 h	2 h	4 h	6 h
**MAP (mmHg)**					
Control	120±1	121±1	122±3	121±3	116±2
LPS1h	120±5	70±3*	–	–	–
LPS2h	121±4	73±4*	107±4*	–	–
LPS4h	117±2	67±5*	105±5*	94±6*	–
LPS6h	117±2	67±4*	101±3*	94±5*	68±4*
**HR (beats/min)**					
Control	363±20	377±3	393±22	401±19	376±17
LPS1h	361±23	430±39*	–	–	–
LPS2h	357±9	443±26*	561±13*	–	–
LPS4h	361±12	418±8*	487±13*	597±18*	–
LPS6h	339±18	391±9*	480±17*	552±15*	561±16*
**ALT (µkat/L)**					
Control	0.40±0.03	0.35±0.03	0.33±0.03	0.33±0.03	0.28±0.03
LPS1h	0.48±0.05	0.70±0.12	–	–	–
LPS2h	0.50±0.10	0.60±0.18	0.80±0.18*	–	–
LPS4h	0.43±0.03	0.68±0.18	0.85±0.30*	4.48±0.62*	–
LPS6h	0.42±0.02	0.52±0.10	0.62±0.08*	3.49±0.55*	13.63±0.58*
**CRE (µmol/L)**					
Control	17.68±0	17.68±0	17.68±0	17.68±0	17.68±0
LPS1h	17.68±0	35.36±2.65*	–	–	–
LPS2h	17.68±0	35.36±2.65*	35.36±2.65*	–	–
LPS4h	17.68±0	35.36±1.77*	44.20±3.54*	70.72±6.19*	–
LPS6h	17.68±0	35.36±2.65*	35.36±2.65*	61.88±4.42*	88.40±3.54*
**BUN (mmol/L)**					
Control	6.42±0.36	6.07±0.36	6.07±0.36	6.42±0.71	6.07±0.36
LPS1h	7.85±0.71	12.85±0.71*	–	–	–
LPS2h	6.42±0.71	11.07±0.71*	13.92±1.07*	–	–
LPS4h	7.50±0.36	11.78±0.71*	13.92±0.71*	19.99±1.07*	–
LPS6h	6.75±0.36	10.71±0.36*	12.85±0.71*	19.26±0.71*	24.99±0.71*
**LDH (µkat/L)**					
Control	1.45±0.15	1.40±0.13	1.25±0.13	1.20±0.10	1.37±0.15
LPS1h	1.79±0.22	7.60±0.89*	–	–	–
LPS2h	1.57±0.17	5.18±0.60*	8.60±1.34*	–	–
LPS4h	1.74±0.12	5.11±0.82*	8.45±2.39*	46.19±16.17*	–
LPS6h	1.65±0.13	4.53±0.38*	6.73±0.58*	47.61±11.79*	128.44±18.57*
**Nitrite/nitrate (µmol/L)**					
Control	21±4	19±4	18±4	27±5	29±6
LPS1h	23±3	25±4	–	–	–
LPS2h	23±4	26±4	25±6	–	–
LPS4h	19±4	22±3	29±5	135±13*	–
LPS6h	23±2	27±5	33±5	200±20*	508±69*

Depicted are changes of mean arterial pressure (MAP), heart rate (HR), alanine aminotransferase (ALT), creatinine (CRE), blood urea nitrogen (BUN), lactate dehydrogenase (LDH) and nitric oxide (NO) in control group (0.9% NaCl, infusion 10 min, *n* = 8) and LPS groups (lipopolysaccharide, LPS 10 mg/kg; infusion 10 min, *n* = 8 in each group). The LPS groups were further divided into 4 groups, which were sacrificed at 1 h after LPS (LPS1h), at 2 h after LPS (LPS2h), at 4 h after LPS (LPS4h) and at 6 h after LPS (LPS6h), separately. Data are expressed as mean ± SEM.

* *P*<0.05 LPS vs. Control.

### Time-course Changes of Pressor Response to NA *in vivo* and *ex vivo*

**Figure 2:**
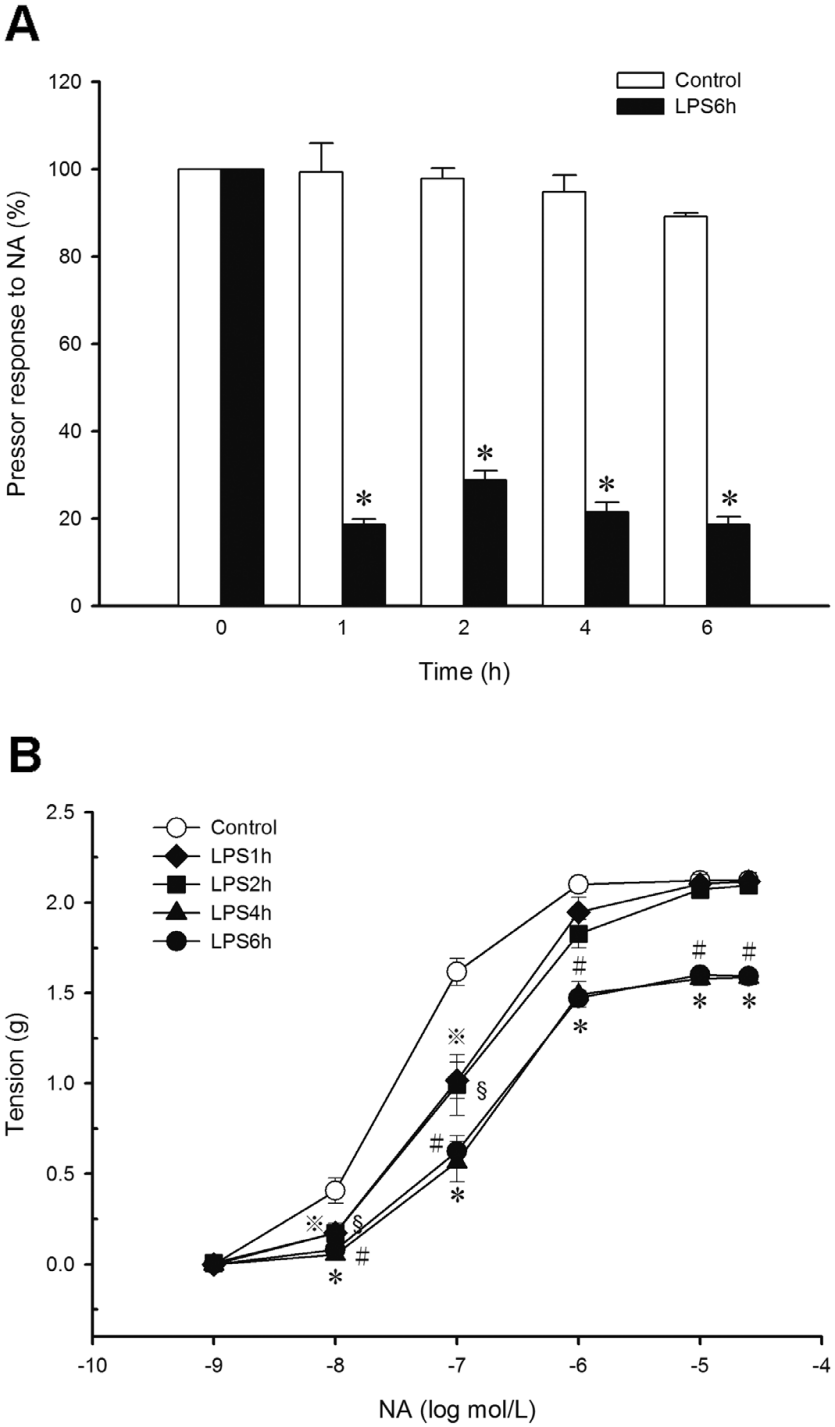
Changes of (A) pressor response to noradrenaline (NA) in rats treated with lipopolysaccharide (LPS) and (B) concentration-response to NA in aortas from rats treated with LPS or saline *ex vivo* are presented. (A) Depicted are changes in pressor response to NA (1 µg/kg, i.v.) during the experimental period in rats that received saline (Control; 0.9% NaCl, infusion 10 min, *n* = 8) and LPS (10 mg/kg, infusion 10 min, *n* = 8). Data are expressed as mean ± SEM. *P<0.05, LPS vs. Control. (B) Depicted are isometric tension recording traces of NA (1 nmol/L – 30 µmol/L)-induced contraction in the aorta from control group (0.9% NaCl, infusion 10 min, *n* = 8) and LPS groups (LPS 10 mg/kg, i.v.; rats were sacrificed at 1 h, 2 h, 4 h or 6 h after LPS, *n* = 8 in each group). Data are expressed as mean ± SEM. ^
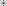
^ *P*<0.05, LPS1h vs. Control; ^§^ *P*<0.05, LPS2h vs. Control; *^#^P*<0.05, LPS4h vs. Control; *P<0.05, LPS6h vs. Control.

The baseline values for pressor response to NA of control and LPS6h groups were not significantly different. In the control group, there was no significant change in NA-induced pressor response during the experimental period. In contrast, animals treated with LPS showed a significant attenuation of pressor response to NA at each time-point (baseline as 100%; 1 h, 18.7±1.3%; 2 h, 28.8±2.2%; 4 h, 21.5±2.2%; 6 h, 18.7±1.8%, *n* = 8, *P*<0.05) when compared to the control (Fig. 2A).

In the *ex vivo* study, NA (1 nmol/L – 30 µmol/L) caused a concentration-dependent contraction in aortas from all groups (control group, *E_max_* = 2.12±0.04 g, EC_50_ = 31.7±5.5 nM; LPS1h group, *E_max_* = 2.12±0.05 g, EC_50_ = 121.1±17.5 nM; LPS2h group, *E_max_* = 2.10±0.05 g, EC_50_ = 154.3±36.8 nM; LPS4h group, *E_max_* = 1.58±0.04 g, EC_50_ = 271.3±41.2 nM; LPS6h group, *E_max_* = 1.59±0.04 g, EC_50_ = 296.4±31.2 nM, *n* = 8). In LPS4h and LPS6h groups, there was a significant attenuation of the contractile response to NA when compared to the control group (*P*<0.05). The EC_50_ were significantly increased in all LPS-treated groups when compared to the control group (*P*<0.05), however, the maximal contractile response to NA was not significantly changed in aortas from LPS1h and LPS2h groups when compared to the control group (Fig. 2B). Interestingly, the hyporeactivity did not occur in early endotoxaemia *ex vivo* which was different from the response *in vivo*.

### Time-course Changes of RhoA Activity, Protein Expression and MYPT1 Phosphorylation at Thr696, Thr850 in the Aorta from LPS-treated Rats

**Figure 3:**
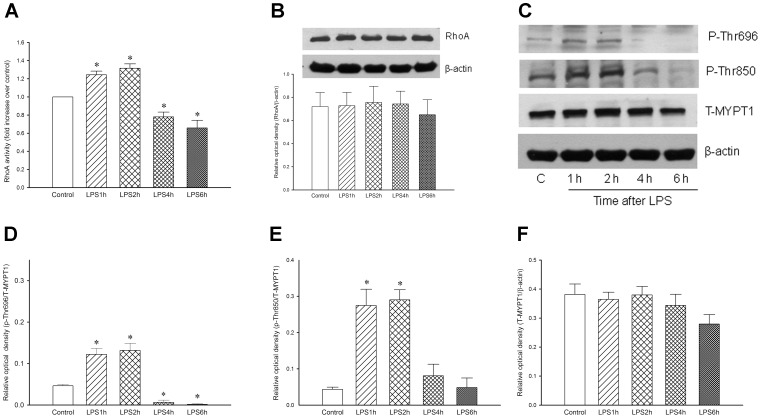
Changes of (A) RhoA activity, (B) RhoA protein expression, and (C) a representative Western blot for phosphorylated-MYPT1 at Thr696, Thr850 and total MYPT1 in aortas from rats treated with lipopolysaccharide (LPS) as well as quantitative analysis of phosphorylated-MYPT1 at (D) Thr696 and (E) Thr850, and (F) total protein expression of MYPT1 in aortas from rats treated with lipopolysaccharide (LPS) are presented. Depicted are changes of RhoA activity and protein expression, phosphorylated-MYPT1 at Thr696, Thr850 and total MYPT1 in the aorta from control group (0.9% NaCl, infusion 10 min, *n* = 3 for RhoA protein expression, *n* = 5 for RhoA activity, *n* = 3 for total MYPT1 and phosphorylated-MYPT1 protein expression) and LPS groups (LPS 10 mg/kg, infusion 10 min; rats were sacrificed at 1 h, 2 h, 4 h or 6 h after LPS, *n* = 3 for RhoA protein expression, *n* = 5 for RhoA activity, *n* = 3 for total MYPT1 and phosphorylated-MYPT1 protein expression). Data are expressed as mean ± SEM. *P<0.05, LPS vs. Control.

To investigate whether the RhoA/ROK pathway was involved in the regulation of vascular reactivity in early endotoxaemia, the activity of RhoA in the aorta was determined in all groups. The administration of rats with LPS for 1 h and 2 h caused a significant increase of RhoA activity (+24% and +32%, *n* = 5, *P*<0.05) when compared to the control. In contrast, this activity was decreased at 4 h and 6 h after LPS (−22% and −34%, *n* = 5, *P*<0.05) when compared to the control group (Fig. 3A). However, the expression of RhoA was not significantly different between the control group and various LPS groups (Fig. 3B).

Simultaneously, we also evaluated the changes of MYPT1 in aortas. MYPT1 phosphorylation sites at Thr696 and Thr850 are downstream targets of RhoA/ROK. The MYPT1 phosphorylation at Thr696 in aortas obtained from LPS1h and LPS2h groups were significantly increased (+140% and +160%, *n* = 3, *P*<0.05) when compared to the control group, whereas its phosphorylation at Thr696 were significantly decreased in aortas from LPS4h and LPS6h groups (−88% and −96%, *n* = 3, *P*<0.05) when compared to the control group (Fig. 3C, 3D). In addition, a significant increase of MYPT1 phosphorylation at Thr850 was observed in LPS1h and LPS2h groups (+600% and +625%, *n* = 3, *P*<0.05) when compared to the control group (Fig. 3C, 3E). However, the total protein expression of MYPT1 was not significantly changed in aortas from all groups (Fig. 3C, 3F). Thus, we suggest that activation of the RhoA/ROK pathway could modulate vascular hyporeactivity occurring in early endotoxaemia.

### Effect of Y27632 on NA-induced Contraction of Aortas in Early Endotoxaemia

**Figure 4:**
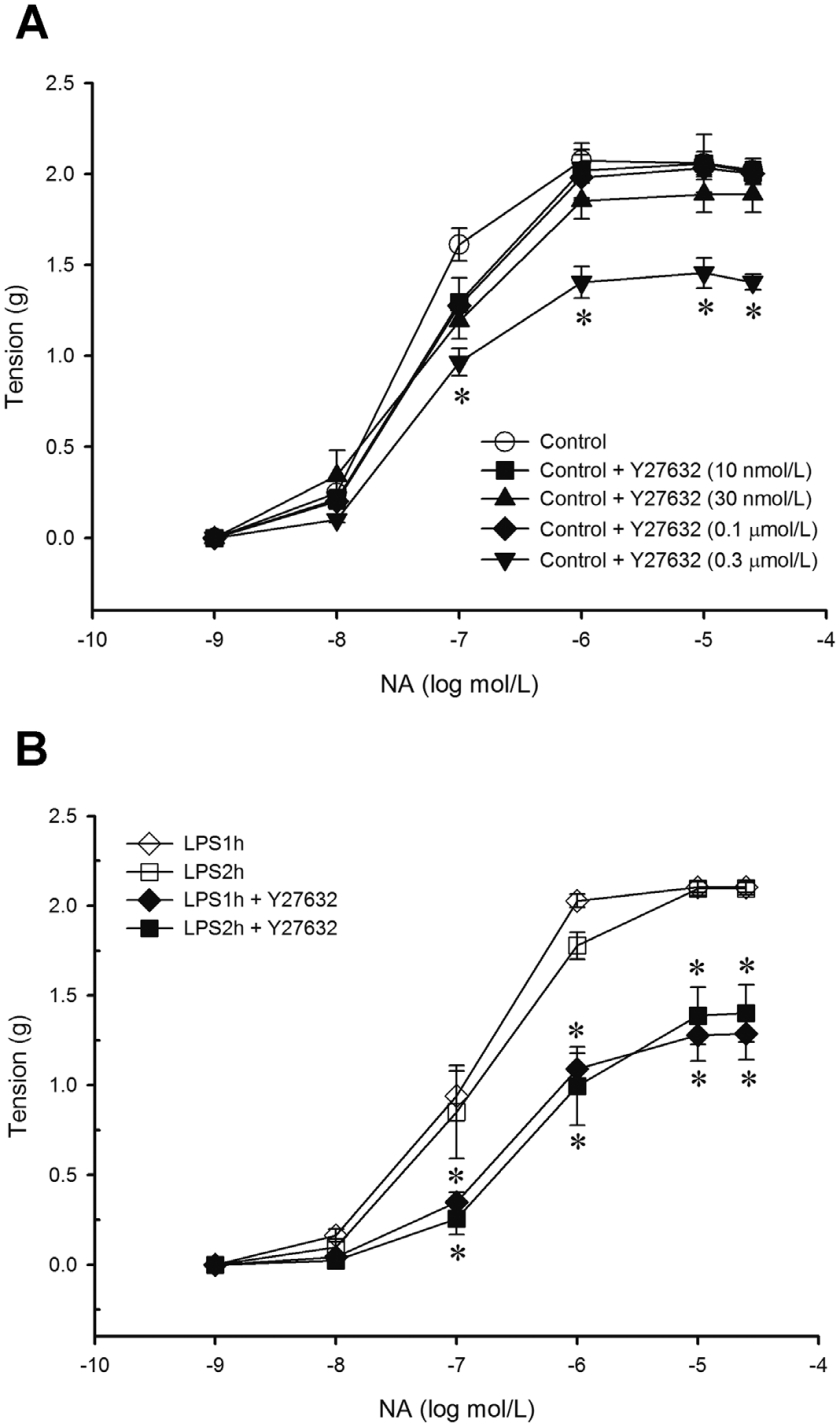
Changes of (A) concentration-response curves of Y27632 on noradrenaline (NA)-induced contraction in aortic rings from the control group and (B) effects of Y27632 on NA-induced contraction in aortic rings from LPS1h and LPS2h groups are presented. (A) Depicted are various concentrations of ROK inhibitor Y27632 (10 nmol/L – 0.3 µmol/L) on NA-induced contraction in aortic rings from the control group (0.9% NaCl, infusion 10 min, *n* = 7 for each groups). (B) Depicted are 0.1 µmol/L Y27632 on NA (1 nmol/L – 30 µmol/L)-induced contraction in aortas from LPS groups (lipopolysaccharide, LPS 10 mg/kg; infusion 10 min; rats were sacrificed at 1 h or 2 h after LPS, *n* = 5 for each group). Data are expressed as mean ± SEM. *P<0.05, with vs. without Y27632.

To further explore our hypothesis that activation of RhoA/ROK pathway may regulate vascular hyporeactivity occurred in early endotoxaemia, a concentration-dependent *ex vivo* study of Y27632 (ROK inhibitor, 10 nmol/L –0.3 µmol/L) on NA-induced contraction was performed in aortic rings from the control group. Results demonstrated that 0.1 µmol/L of Y27632 is the maximal concentration which did not affect vascular tension (Fig. 4A). Thus, 0.1 µmol/L of Y27632 was used to examine the NA-induced contraction in aortas from LPS1h and LPS2h groups. Results demonstrated that Y27632 caused a significant reduction of NA-induced contraction in aortas from LPS1h (*E_max_* from 2.10±0.03 g to 1.29±0.14 g, *n* = 5, *P*<0.05) and LPS2h groups (*E_max_* from 2.09±0.04 g to 1.40±0.16 g, *n* = 5, *P*<0.05) when compared to the control group (Fig. 4B). Indeed, our results support the hypothesis that activation of RhoA/ROK pathway acts to compensate vascular hyporeactivity occurring in early endotoxaemia.

### Effects of Y27632 in Haemodynamic Change with Endotoxaemia

Based on the results of Fig. 3 and 4, we tested the effects of Y27632 in haemodynamic change with endotoxaemia. The animals in LPS group reproduced a biphasic hypotension also followed by a significant increase in HR during the experimental period, and sustained attenuated pressor responses to NA when compared to the baseline. In Y27632+LPS group, Y27632 itself caused a rapid fall in MAP (72±2 mmHg at 15 min after Y27632), which was accompanied by a significant increase in HR (449±29 beats/min at 15 min after Y27632) when compared to the baseline, and then the MAP recovered to baseline at 1 h after Y27632. After rats challenged with LPS, the MAP rapid fell again and had a significant decrease, and a significant increase in HR at 2 h after LPS when compared to the LPS group (*n* = 5, *P*<0.05; Table 2). Thereafter, rats died within 4 h after LPS injection.

**Table 2:** Effects of Y27632 on haemodynamic changes in rats with endotoxaemia.

	Baseline	Post-LPS (1 h)	Post-LPS (2 h)
**MAP (mmHg)**			
LPS	122±2	76±3*	103±2*
Y27632+LPS	120±2	73±3*	89±5* ^,^ #
**HR (beats/min)**			
LPS	357±14	450±23*	508±15*
Y27632+LPS	352±14	462±19*	534±13*
**Pressor response to NA (%)**			
LPS	100	27.69±2.37*	26.28±1.82*
Y27632+LPS	100	19.46±2.62* ^,^ #	19.65±1.20* ^,^ #

Depicted are the changes of mean arterial pressure (MAP), heart rate (HR) and pressor response to NA in LPS group (lipopolysaccharide, LPS 10 mg/kg; infusion 10 min, *n* = 5) and Y27632+LPS group (Y27632 5 mg/kg followed by LPS 10 mg/kg, infusion 10 min, *n* = 5). Data are expressed as mean ± SEM.

* *P*<0.05, each time-point compared to baseline in identical group;

# *P*<0.05, Y27632+LPS group compared to LPS group at the same time.

In addition, Y27632 itself also caused significant attenuation of the pressor response to NA (45.95±3.6% at 1 h after Y27632) when compared to the baseline. Furthermore, significant decreases at 1 h and 2 h after LPS were observed when compared to the LPS group (*n* = 5, *P*<0.05) (Table 2).

### Time-course Changes of eNOS Protein Expression in the Aorta and Plasma BK Level from LPS-treated Rats

**Figure 5:**
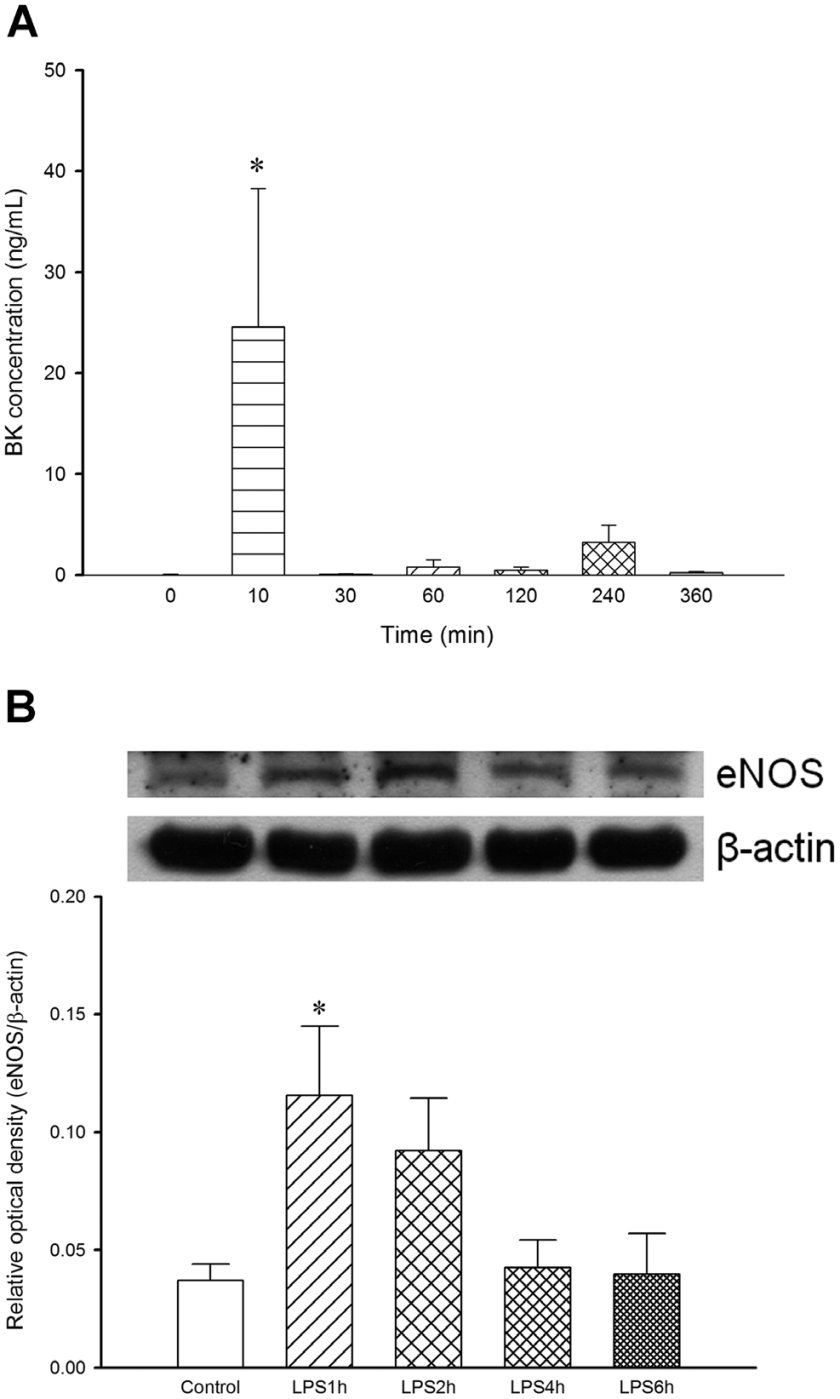
Changes of (A) plasma bradykinin (BK) level in rats before and after treatment with lipopolysaccharide (LPS) and (B) endothelial nitric oxide synthase (eNOS) protein expression in aortas from rats treated with LPS are presented. Depicted are changes of plasma BK level during the experimental period in rats before and after treatment with LPS (LPS 10 mg/kg, infusion 10 min, *n* = 3–5), and eNOS protein expression in aortas from control group (0.9% NaCl, infusion 10 min, *n* = 3) or LPS groups (LPS 10 mg/kg, infusion 10 min.; rats were sacrificed at 1 h, 2 h, 4 h or 6 h after LPS, *n* = 3). Data are expressed as mean ± SEM. *P<0.05, (A) vs. time 0 and (B) vs. Control.

To investigate the mechanism of early hypotension induced by LPS, we measured the plasma BK level and eNOS expression in aortas. Results demonstrated that the plasma BK level was undetectable in rats before LPS treatment. A transient increase in the plasma BK level at 10 min after LPS (24.6±13.7 ng/mL, *n* = 5, *P*<0.05) was observed when compared to the baseline (Fig. 5A). In addition, a significant increase of eNOS expression was also observed in the LPS1h group (+200%, *n* = 3, *P*<0.05), whereas there was no significant change of eNOS in LPS4h and LPS6h groups when compared to the control group (Fig. 5B). This indicates that endogenous BK and eNOS are involved in early hypotension induced by LPS, which may also contribute to vascular hyporeactivity to NA *in vivo*.

### Time-course Changes of Serum NO Level and Aortic iNOS Protein Expression

**Figure 6:**
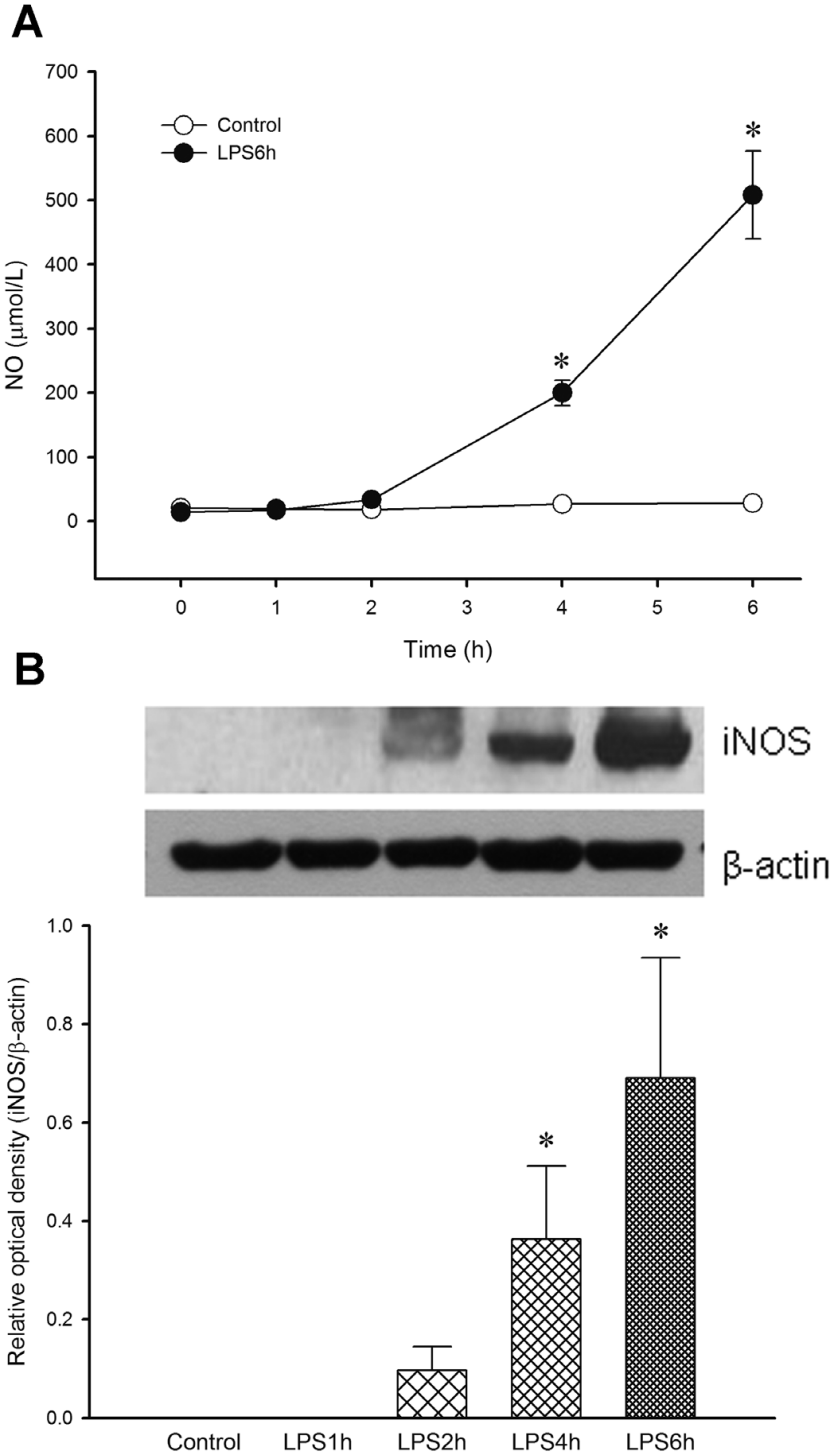
Changes of (A) serum nitric oxide (NO) level and (B) inducible nitric oxide synthase (iNOS) protein expression in aortas from rats treated with lipopolysaccharide (LPS) are presented. Depicted are changes in serum level of NO and aortic iNOS expression during the experimental period in rats received saline (Control; 0.9% NaCl, infusion 10 min, *n* = 8 for NO level and *n* = 3 for iNOS expression) or LPS (LPS 10 mg/kg, i.v.; rats were sacrificed at 1 h, 2 h, 4 h or 6 h after LPS, *n* = 8 for NO level and *n* = 3 for iNOS expression). Data are expressed as mean ± SEM. *P<0.05, LPS vs. Control.

Fig. 3A shows that RhoA activity is inhibited in late endotoxaemia. It has been shown that cGMP/PKG pathway can inhibit RhoA activity and cause vasodilation [29]. Therefore, we determined changes of serum NO level and aortic iNOS protein expression in LPS-treated rats. There was no significant change in serum NO level in the control group, whereas LPS caused a time-dependent elevation in serum NO level (at 6 h, 508±69 µmol/L, *n* = 8, *P*<0.05) when compared to the control group (Fig. 6A).

The protein expression of iNOS was undetectable in aorta homogenates obtained from control and LPS1h groups, whereas a significant expression of iNOS was observed in aorta homogenates obtained from LPS4h and LPS6h groups (Fig. 6B). These data indicate that RhoA activity is inhibited by NO produced from iNOS in late endotoxaemia.

## Discussion

**Figure 7:**
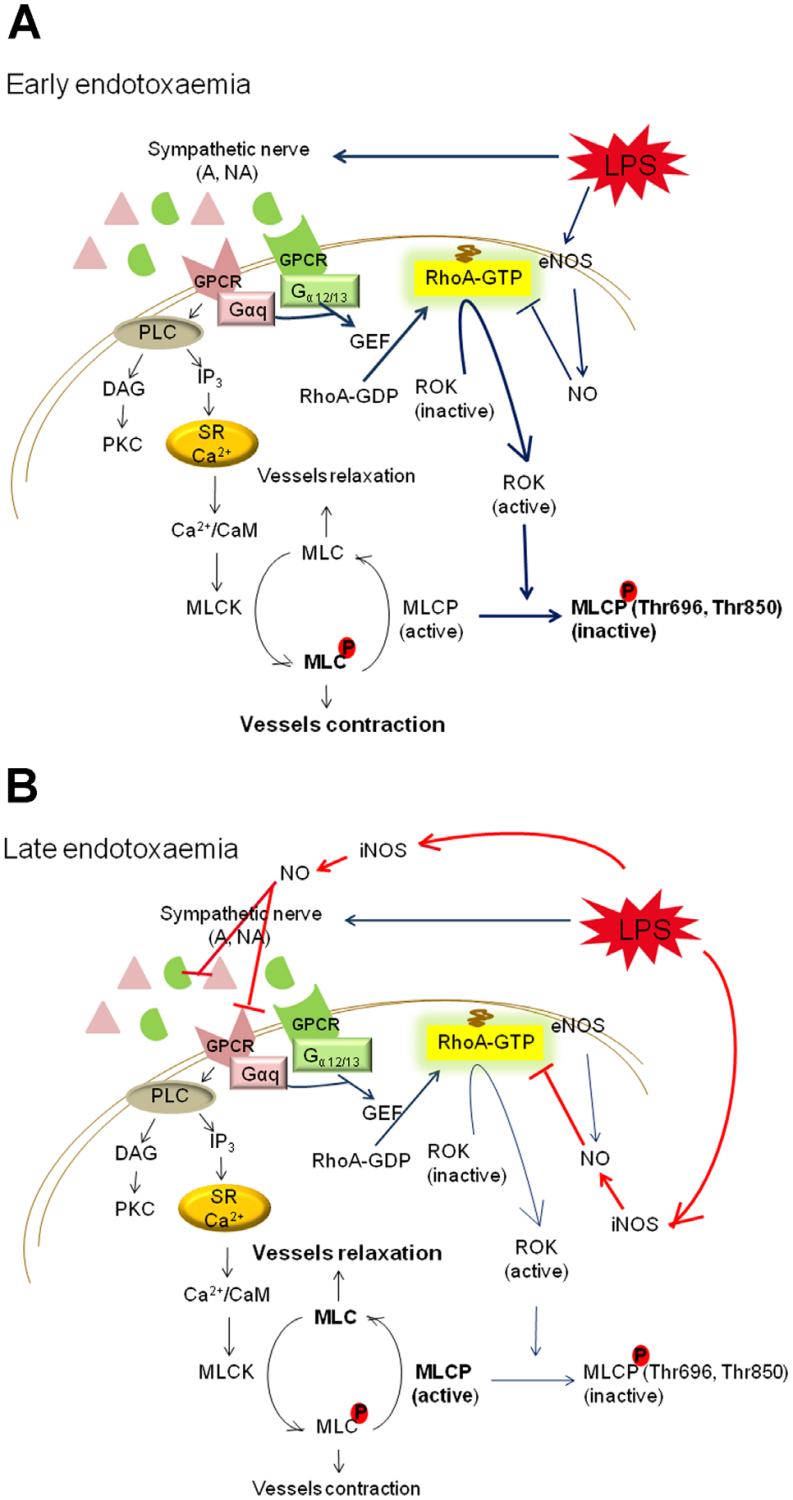
RhoA/ROK pathway is (A) activated in early endotoxaemia (blue line) and (B) inhibited in late endotoxaemia (red line). LPS, lipopolysaccharide; GEF, guanine nucleotide exchange factor; ROK, Rho-kinase; NO, nitric oxide; eNOS, endothelial nitric oxide synthase; iNOS, inducible nitric oxide synthase; GPCR, G protein-coupled receptor; PLC, phospholipase C; DAG, diacylglycerol; PKC, protein kinase C; IP_3_, inositol 1,4,5-triphosphate; SR, sarcoplasmic reticulum; CaM, Ca^2+^/calmodulin; MLCK, myosin light-chain kinase; MLCP, myosin light-chain phosphatase; A, adrenaline; NA, noradrenaline.

In the present study, LPS (i) induced a biphasic hypotension, (ii) caused a sustained decrease of the pressor response to NA *in vivo* and vascular hyporeactivity to NA *ex vivo* in late endotoxaemia only, (iii) increased aortic RhoA activity and MYPT1 phosphorylation to maintain pressor response to NA and vascular reactivity which was inhibited by Y27632, and transiently increased plasma BK level and aortic eNOS expression in early endotoxaemia, and (iv) increased the serum NO level associated with aortic iNOS expression and decreased aortic RhoA activity and MYPT1 phosphorylation in late endotoxaemia. These findings suggest that the vascular reactivity in early endotoxaemia is maintained by activation of the RhoA/ROK pathway (to counteract the early hypotension) (Fig. 7A) which is suppressed by iNOS-induced NO in late endotoxaemia, leading to vascular hyporeactivity both *in vivo* and *ex vivo* (Fig. 7B).

Interestingly, our results showed that administration of rats with LPS induced a significant attenuation of pressor response to NA from 1 h to 6 h *in vivo,* but the maximal response to NA in aortic rings from LPS1h and LPS2h groups was not significantly different from that in the control group. In addition, the aortic RhoA activity and phosphorylated MYPT1 at Thr696 and Thr850 were increased in early endotoxaemia. The *in vivo* experiments with the ROK inhibitor Y27632 were performed and the pressor response in early endotoxemia was inhibited by Y27632. Previous studies have demonstrated that the activity of the sympathetic nervous system is augmented in early endotoxaemia [30], and increased levels of catecholamines (including adrenaline and NA) are released from the adrenal medulla in animals treated with LPS [31]. It is possible that increased endogenous NA binding to α_1_-adrenergic receptor activates Gαq and Gα_12/13_, and hence, increases the activity of RhoA [2]. This explicates that why the vascular reactivity could be maintained and indicates that RhoA/ROK pathway is involved in the regulation of vascular reactivity in early endotoxaemia.

It has been shown that the immediate transient hypotension induced by LPS is mainly associated with the release of endogenous BK, which activates eNOS, resulting in vasodilation [21]–[23]. Indeed, in this study, we also demonstrated that administration of rats with LPS showed a significant increase in the plasma level of BK. Although the expression of eNOS was increased in the LPS1h group, NO produced by eNOS seems insufficient to attenuate the vessel tone which is maintained by increased RhoA activity in early endotoxaemia. It has been reported that chronic eNOS overexpression in the endothelium of mice results in resistance to LPS-induced hypotension and death, and these effects are attributed to the reduced response to NO [32]. Our results showed that overexpression of eNOS only appeared in early endotoxaemia, and returned to basal level in late endotoxaemia. This may be one of the possibilities why animals did not have resistance to LPS-induced late hypotension and death. However, the role of eNOS in early endotoxaemia needs to be further clarified.

At 2 h after LPS, the MAP fell again and continued to decline in the rest of experimental period. Moreover, in late endotoxaemia, a significant reduction of the vascular response to NA was observed in both *in vivo* and *ex vivo* studies, and activity of RhoA and phosphorylation of MYPT1 in aortas were also decreased. Experimental and clinical studies suggest that excessive NO production by iNOS is responsible for hyporesponsiveness to NA and hypotension in endotoxaemia [19], [24], [25]. In this study, we demonstrated that both aortic iNOS expression and serum NO level were significantly increased in late endotoxaemia. A large amount of NO produced by iNOS may cause (i) desensitization of α_1_-adrenoceptor [33], (ii) deactivation of NA [34], and (iii) inhibition of RhoA/ROK pathway via a Ca^2+^-desensitization mechanism [12], [13]. Here, we showed that both activity of RhoA and phosphorylation of MYPT1 at Thr696 and Thr850 were decreased at 4 h and 6 h after LPS, indicating that in late endotoxaemia, the high-output NO production by iNOS could inhibit RhoA/ROK pathway and lead to vascular hyporeactivity, systemic hypotension, and organ dysfunction (e.g. increases of ALT, CRE, BUN and LDH levels in this study). Thus, our *in vivo* data further confirmed that in late endotoxaemia, the RhoA/ROK pathway could participate in the pathogenesis of vascular hyporeactivity accompanied with endotoxaemic shock.

Taken together, this study demonstrated that the injection of rats with LPS induced a biphasic hypotension and a decreased pressor response to NA. Importantly, an increased RhoA activity may compensate vascular reactivity in early endotoxaemia *ex vivo* (Fig. 7A), while in late endotoxaemia, the large production of NO by iNOS inhibiting RhoA activity leads to vascular hyporeactivity *in vivo* and *ex vivo* (Fig. 7B). Therefore, both RhoA/ROK and iNOS/NO pathways play important roles in regulation of vascular reactivity and blood pressure in endotoxaemic rats. We propose that a combination of using NO/cGMP pathway inhibitors and specific RhoA activators could be a novel therapeutic strategy in the vascular hyporeactivity associated with endotoxaemic shock.
